# Deep sequencing reveals global patterns of mRNA recruitment during translation initiation

**DOI:** 10.1038/srep30170

**Published:** 2016-07-27

**Authors:** Rong Gao, Kai Yu, Jukui Nie, Tengfei Lian, Jianshi Jin, Anders Liljas, Xiao-Dong Su

**Affiliations:** 1 Biodynamic Optical Imaging Center (BIOPIC), and State Key Laboratory of Protein and Plant Gene Research, School of Life Sciences, Peking University, Beijing 100871, China; 2 Department of Biochemistry and Structural Biology, Lund University, Lund, Sweden

## Abstract

In this work, we developed a method to systematically study the sequence preference of mRNAs during translation initiation. Traditionally, the dynamic process of translation initiation has been studied at the single molecule level with limited sequencing possibility. Using deep sequencing techniques, we identified the sequence preference at different stages of the initiation complexes. Our results provide a comprehensive and dynamic view of the initiation elements in the translation initiation region (TIR), including the S1 binding sequence, the Shine-Dalgarno (SD)/anti-SD interaction and the second codon, at the equilibrium of different initiation complexes. Moreover, our experiments reveal the conformational changes and regional dynamics throughout the dynamic process of mRNA recruitment.

Translational control is an important type of posttranscriptional regulation in determining weather, when, and how much protein will be synthesized with a given mRNA^1^. Regulation at the translational level accounts for large variations in expression among different genes^2^. Among the many aspects that can regulate translation efficiency^2^,^3^,^4^,^5^, translation initiation is usually considered a key determinant of the translational yield for most mRNAs^6^,^7^,^8^,^9^.

The translation initiation efficiency of a given mRNA is determined by its translation initiation region (TIR)^1^. TIRs have varied sequences with some preferred bases, but the sequences are non-unique^9^,^10^. A combination of multiple elements in this region is usually thought to contribute to the mRNA recruitment^11^, which includes the initiation codon, the Shine-Dalgarno (SD) sequence, the availability of the SD sequence for binding to the anti-SD (ASD) sequence near the 3′ end of the 16S rRNA^8^, the distance between the SD sequence and the initiation codon, and the specific enhancer sequences (A/U-rich elements) recognized by protein S1 of the 30S ribosomal subunit^7^,^11^. The efficiency of mRNA recruitment is the cooperative and cumulative result of the multiple elements at the translation initiation region^9^,^10^.

Translational initiation is a dynamic process and is often referred to as the rate-limiting step of translation^3^,^12^,^13^. In bacteria, the binding of mRNAs and formation of the translational initiation complex mainly proceed in three stages: assembly of the 30S pre-initiation complex (30S PIC), transition into a mature 30S initiation complex (30S IC) with structural rearrangements, and final formation of the 70S initiation complex (70S IC) that is ready for elongation^11^,^13^,^14^,^15^. Initial binding of mRNA has been reported independent of any initiation factors and can take place at any moment during 30S PIC assembly^16^. This rapid binding step facilitates the swift recruitment of mRNAs to the 30S subunit of the ribosome, but with poor specificity^8^,^17^. To guarantee that translation starts at the proper site, mRNA binding to the ribosome depends on kinetic control based on multiple checkpoints as the initiation complex proceeds from the 30S PIC to the 30S IC and finally to the 70S IC^11^,^16^.

Although extensive research has been carried out to study the effect of the initiation elements, the focus has mainly been on the manipulation of protein expression by the 70S IC, the final stage of the initiation complex. Moreover, although many experiments have been performed with a single RNA sequence to study the kinetics of mRNAs at different stages of initiation^1^,^7^,^8^,^11^,^16^, such experiments cannot easily reveal the entire range of sequence possibilities for mRNA recruitment. In this study, we attempted to elucidate the dynamic process of mRNA recruitment and to study the selection of mRNAs at a truly global level. We developed techniques based on high-throughput sequencing (deep sequencing) to clarify the behavior of the potential initiation elements at the equilibrium state in different stages of initiation complexes (Supplementary Fig. S1). To achieve this goal, artificial mRNA libraries were prepared with a randomized region at desired positions (Supplementary Fig. S2). These mRNA libraries were selected by the 30S ribosome, the 30S IC, and the 70S IC individually (referred to as 30S, 30SIC, and 70SIC from here on). Our results provide a comprehensive view of the dynamic process of mRNA recruitment during translation initiation.

## Results

As shown in Supplementary Figs S1 and S2, we used different mRNA libraries. Our experiments were carried out at equilibrium conditions. Briefly, each mRNA library was incubated with ribosomes (30S, 30SIC, or 70SIC) at 37 °C for 30 minutes to form mRNA-ribosome complexes. The bound RNAs were then separated from the unbound RNAs by binding His-tagged ribosomes to Ni-NTA resin with additional incubation at 4 °C for 16 hours.

### Overview of the sequence properties in the TIR upstream of AUG

**Figure 1:**
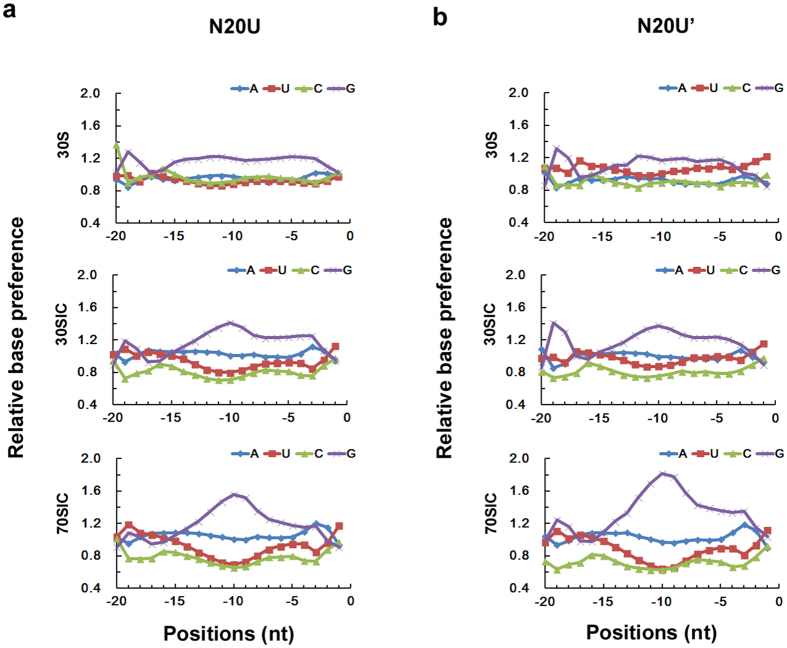
Overall view of the base preference for mRNA library N20U with 20 randomized nucleotides in the TIR upstream of AUG. (**a**,**b**) Results from two independent repeats. In each repeat, the base preference was illustrated in three translation initiation stages: 30S, 30SIC and 70SIC.

To elucidate the sequence properties in the TIR upstream of AUG (−20 to −1), we synthesized the mRNA library N20U, which contains 20 randomized nucleotides upstream of the AUG codon (Supplementary Fig. S2). The mRNA library was selected by the 30S subunit, the 30SIC or the 70SIC, followed by deep sequencing with a non-selected mRNA library as the blank control (Supplementary Table S2). The results from two independently repeated experiments are provided in Fig. 1. The two repeats presented similar base preference in the TIR upstream of AUG. After determining the influence of the background library (with the relative value normalized to 1 for the background), we detected striking base preferences at different positions during the process of forming different initiation complexes. Obviously, this is a dynamic process with the initiation signals accumulated to different extents at different stages. A significant enrichment in G-bases in the middle region of the randomized 20-base region (−15 to −5) was observed during 30SIC and 70SIC formation, but not so obviously in 30S alone. U-bases were preferred at the beginning of the randomized region (−20 to −15) and probably also at the region close to the AUG codon, but not at the middle region (−15 to −5). A-bases were mildly selected all over the region (−20 to −1). Compared with other bases, C-bases were poorly selected in the randomized region of N20U, in agreement with a previous report^18^. The observed base preference provided a global view of the cumulative elements in this region, including the A/U-rich sequences at the 5′ end of the mRNA and the A/G-rich SD sequences. In addition, the mildly accumulated A-bases throughout the region might imply a requirement for a loose secondary structure in this region. It does not seem accidental that we observed a small peak in A-bases in the −3 position, followed by the increased preference for U- and C-bases in the next two positions (−2 and −1).

### Codon preference for the second codon

**Figure 2:**
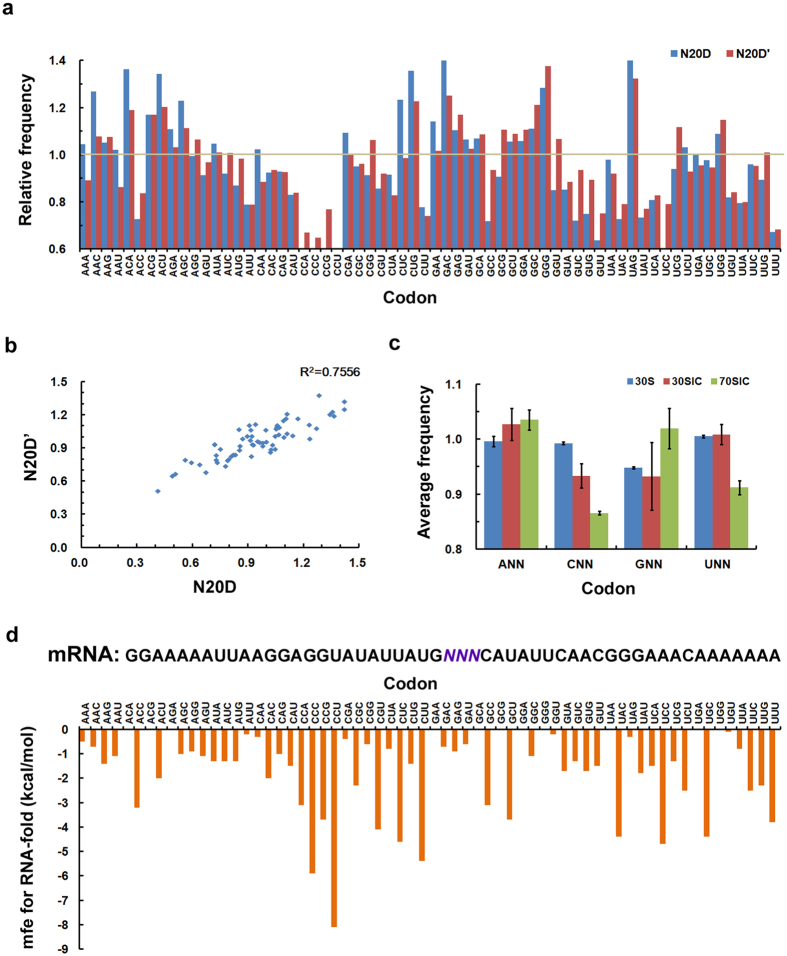
Codon preference for the codon directly following AUG. (**a**) Relative frequency of each of the 64 codons in the second position in the 70SIC. N20D and N20D’ are two independent repeats of the translation initiation experiments. The relative frequency of each of the 64 codons was normalized to frequency per million reads (FPM) and then divided by the frequency from the blank control. The codon is referred to as the preferred codon when the relative codon frequency is higher than 1 (highlighted by the gray line). (**b**) Correlation of the codon preference in the 70SIC of the second codon in two independently repeated experiments. (**c**) The average frequencies of codons ANN, CNN, GNN and UNN in different initiation complexes. The average frequency was the average of the relative frequencies (see definition in **a**) for codons starting with A, C, G and U. The error bar corresponds to the standard deviation of the two repeated experiments. (**d**) The minimum free energy of the structured mRNA differentiated at the second codon after ATG.

The sequence properties were also analyzed at the N-terminus of the translational region (+1 to +20) using mRNA library N20D (D for downstream of AUG). Our sequencing results identified the codon preference for the codon directly following AUG from two independently repeated experiments for N20D. The relative codon frequency selected was analyzed for all 64 codons, and a preference for ANN and GNN in 70SIC was observed (Fig. 2). Figure 2b shows that these two independent experiments were replicated relatively well, with R^2^ equal to 0.7556. A preference for ANN and GNN in 70SIC was also found previously^19^ (Supplementary Fig. S3a,b), even though no distinct correlation could be recognized between our results and the published codon usage^19^ (Supplementary Fig. S3c). The minimum free energy was calculated for the mRNAs containing different codons at the second codon (Fig. 2d). Interestingly, those mRNAs containing the preferred ANN or GNN at the second codon had a more reduced secondary structure, which emphasizes the correlation between the codon preference and the secondary structure of the mRNA in the formation of the initiation complexes^20^,^21^,^22^,^23^. Because our experiments were carried out without any factors involved in the following elongation step, the codon preference at the second codon implies an intrinsic evolutionary requirement from translation initiation. The relative flexible structure around the start codon could help to smooth the initiation and speed up the rate of translation during the first cycle of elongation.

### The sequence properties in the region −20 to −13

**Figure 3:**
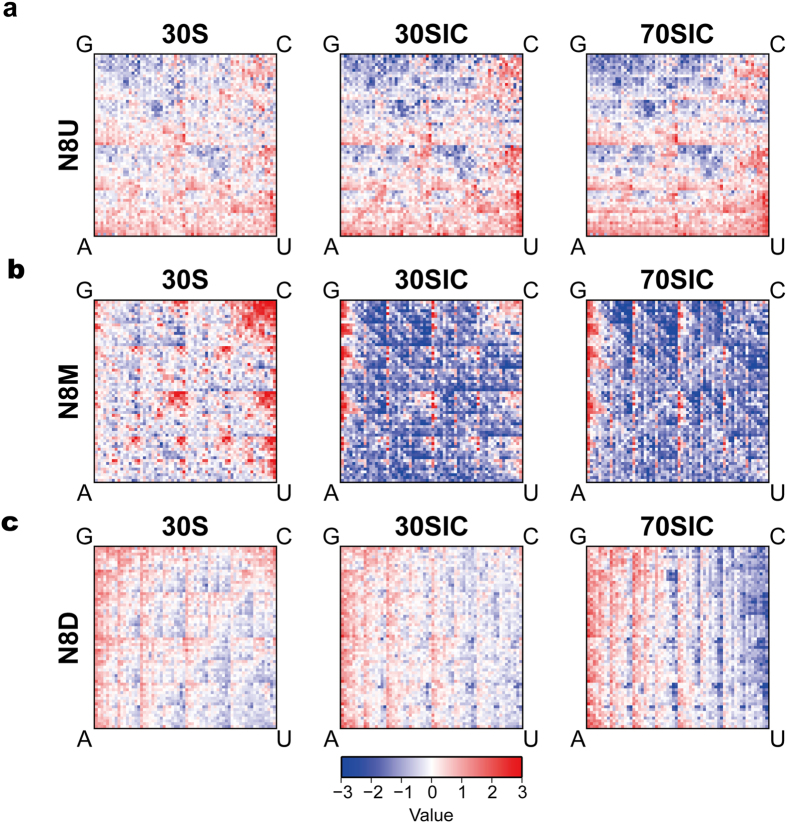
The regional characteristics of the sequence patterns in the TIR upstream of AUG. The k-mer analysis was performed to describe the sequence pattern of each mRNA library, (**a**) N8U, (**b**) N8M and (**c**) N8D, selected by different initiation complexes: 30S, 30SIC and 70SIC. As described in the Methods, each 6-mer sequence has a unique coordinate in the 2^6^ × 2^6^ matrix. At each unique coordinate, the red color means that the 6-mer sequence occurred more frequently than in the background (the sequence occurrence of the same 6-mer in the mRNA library before any selection), whereas the blue color means the 6-mer occurred less frequently than in the background. The value in the color bar describes the extent of the difference in frequency.

To further investigate the regional characteristics of the TIR upstream of AUG, the randomized nucleotides were subdivided into N8U (U for upstream of AUG) (−20 to −13), N8M (−14 to −7) and N8D (−8 to −1). Each of the libraries was selected by the 30S, the 30SIC and the 70SIC. The characteristics of the randomized region in each of the initiation complexes were identified by k-mer analysis (described in the Methods). In our experiment, the k-mer count was evaluated as the sequence occurrence of each of the individual 6-mer sequences in the blank control (the mRNA library alone), 30S, 30SIC, and 70SIC. Then, the influence of the background was deducted from each library by comparison with the blank control. The frequency of the occurrence (KPM_relative_) of each 6-mer was then fitted into a 2D-histogram heat map, which reflected the characteristics of the randomized region enriched during our selection (Fig. 3).

For the mRNA library N8U, a preference for A- and U- bases was identified for the randomized region (Fig. 3a). This sequence pattern is similar to that of the genome analysis of the Gram-negative *Escherichia coli*, but different from the Gram-positive *Bacillus subtilis* (Supplementary Fig. S4, ref. G8U). These observations demonstrate the importance of the ribosomal protein S1 in mRNA recruitment, particularly for the Gram-negative bacteria when the SD/anti-SD interaction is missing^24^,^25^. The presence of S1 is correlated with recognition of the correct start codon through high-affinity binding to an A/U-rich sequence upstream of the start codon, named the translation enhancer^26^,^27^. In the Gram-positive bacterium *B. subtilis* because the S1 protein is missing^28^, the sequence pattern in this region is completely different, with a tendency for A/G preference (Supplementary Fig. S4b, ref. G8U).

Further comparison of the sequence patterns demonstrated similar sequence patterns among three of the initiation stages for N8U (Fig. 3a). Little difference was observed for base preference among the three stages in our experiment. This observation reflects the role of S1 protein in recognizing the mRNA as an anchoring point from the first step in mRNA binding to the 30S subunit^7^. This “anchoring point” was not changed during the later processes when forming the 30SIC and the 70SIC.

### The sequence properties in the region −14 to −7

Our deep sequencing results demonstrated an extraordinary base preference for purines (A- and G-bases), particularly in the region −14 to −7 of mRNAs (Figs 1 and 3b). As the formation of the initiation complexes proceeded from the 30S to the 30SIC, the preference for A/G-bases significantly increased (Figs 1 and 3b). However, there was not much difference in base preference between 30SIC and 70SIC. This observation suggests that there is an mRNA structural rearrangement during the maturation of the 30SIC^8^,^11^.

There was an apparent enrichment of CU in the k-mers for N8M in the 30S. This signal was striking as it was completely reversed in 30SIC and 70SIC. Supplementary Fig. S5 shows the gene pattern representing the abundance of the 6-mer sequences in the reverse complementary sequences (RCS) of the 16S rRNA. A tendency for a preference for C-bases could be observed in the gene pattern of 16S rRNA RCS, which implies a model of rapid binding of the mRNAs to the 30S ribosome by bases complementary to the 16S rRNA during the first stage of mRNA recognition. In contrast to the observations in N8U, the sequence pattern in N8M (70SIC) (Fig. 3b) was similar to those of the genome sequence in the Gram-negative *E. coli* and the Gram-positive *B. subtilis* (Supplementary Fig. S4). The genome sequences all presented a significant preference for A/G-bases in the region −14 to −7, which suggested the common important role of the Shine-Dalgarno sequence in mRNA recruitment in both Gram-positive and Gram-negative bacteria. In the Gram-negative species, however, an alternative S1 mechanism is also provided for mRNA recognition.

### The sequence properties in the region −8 to −1

The study of the mRNA library in the region −8 to −1 also illustrated a sequence preference for A and G bases, although not as distinct as the preference observed in the region of −14 to −7 (Fig. 1). When we compared the sequence patterns of N8D and N8M in different initiation complexes, a selection for sequences rich in C-bases was identified in the 30S complex, but this faded as the initiation complex proceeded to the 30SIC and the 70SIC (Fig. 3c). This observation implies that mRNAs containing C-bases might be selected by a rapid and nonspecific binding to 16S rRNA during the formation of the pre-initiation complex, but will be switched out more easily in the following maturation step, accompanied by the mRNA conformational change.

### Shine-Dalgarno (SD) sequences

**Figure 4:**
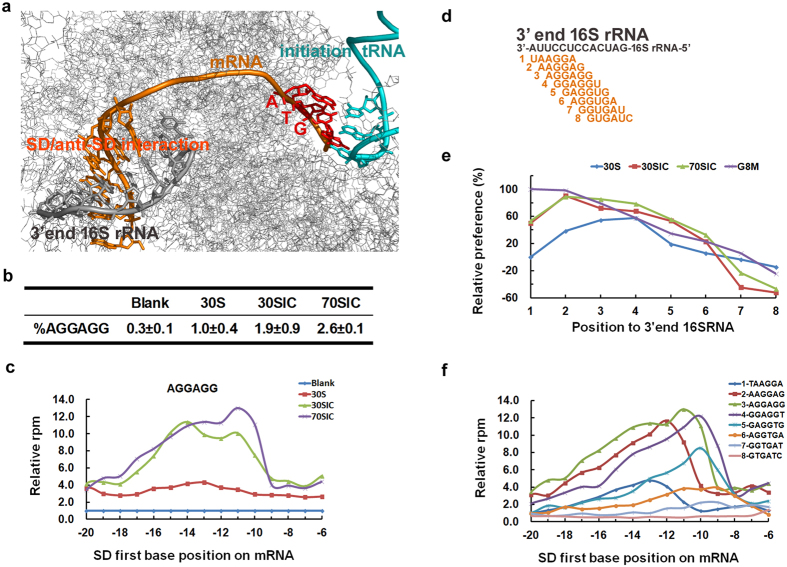
Properties of Shine-Dalgarno (SD) interactions. (**a**) Crystal structure of translation initiation complex (2QNH) illustrating the interactions between SD and anti-SD sequences near the 3′-end of the 16S rRNA as well as their relative position to the initiation codon AUG. The picture was generated using PyMOL (Version 0.98, DeLano Scientific LLC). (**b**) Illustration of the percentage of AGGAGG in N20U in the three initiation stages. (**c**) Illustration of the regional preference of AGGAGG in N20U in the three initiation stages. (**d**) Schematic illustration of the different complementary positions of the SD/anti-SD interactions near the 3′ end of the 16S rRNA. (**e**) Correlation of the relative preference of the eight 6-mers (as indicated in **d**) with their complementary positions at the 3′-end of 16S rRNA. The relative preference was calculated as the percentage of the KPM_relative_ of the target 6-mer compared with the maximum KPM_relative_ from the same selected library (see Fig. 3b and Supplementary Fig. 4a). (**f**) Illustration of the regional preference in N20U in 70SIC for those SD sequences complementary to different positions near the 3′ end of 16S rRNA.

Conventional translation initiation in bacteria involves binding of the 30S ribosomal subunit to the ribosome-binding site (RBS) of an mRNA. This binding is dependent on the purine-rich Shine-Dalgarno sequence, which base-pairs with the anti-SD sequence at the 3′ end of the 16S rRNA to guide the selection of the correct start codon (Fig. 4a)^29^,^30^. This interaction region is normally variable in length and location in relation to the initiation codon^28^, and the strength of the SD-anti SD interaction is part of the equilibrium constant for the binding of mRNA to the 30S subunit^31^.

Our deep sequencing results demonstrated an extraordinary base preference for purines (A- and G-bases), particularly in the region −14 to −7 of the mRNA (Figs 1 and 3b). Using the abundant sequencing results, we investigated the SD properties in more detail. At first, by using AGGAGG, we observed the dynamic accumulation of SD sequences in N20U during mRNA recruitment. The percentage of the mRNAs containing AGGAGG progressively increased from 30S to 30SIC to 70SIC (Fig. 4b,c). Figure 4c further illustrates the regional preference of AGGAGG in mRNAs. The first A-residue of AGGAGG was mainly located at positions −17 to −10 of the mRNAs, which implies the distance between AGGAGG and the initiation codon varied from 4 to 11 bases. This observation is consistent with the preferred position of the SD sequence reported previously^32^. The energy calculation demonstrated that the secondary structure was more stable for those mRNAs with AGGAGG located at the preferred positions (−13 to −10) (Supplementary Fig. S6). This observation implies that a better SD position in an mRNA can compensate for the energy requirement in unfolding the mRNA during initiation.

Further analysis focused on the regional preference of the SD/anti-SD interaction on the 3′ end of 16S rRNA. Figure 4d describes eight 6-mer sequences complementary to different positions on the 3′ end of 16S rRNA. Position number 1 to 8 was defined as the location of the first base on the 3′ end of 16S rRNA. The sequence preference for these eight 6-mer sequences was closely related to their position complementary to the 3′ end of the 16S rRNA (Fig. 4e). Higher regional preference was observed for the sequence complementary closer to the 3′ end of the 16S rRNA. Even though the sequence preference was not closely correlated with their binding affinity for these eight sequences (Supplementary Fig. S7), statistical analysis demonstrated a correlation between the minimum hybridization energy and the sequence preference when the variants of the 6-mer sequences were extended to all the possibilities (4^6^ = 4096) (Supplementary Fig. S8). The tendency of this preference was more notable as the initiation complexes proceeded from the 30S (R^2^ = 0.0016) to the 30SIC (R^2^ = 0.13) to the 70SIC (R^2^ = 0.22).

Additional analysis was devoted to achieving a more general description of the regional preference of the SD/anti-SD interaction on both of the mRNA and the 3′ end of the 16S rRNA. The distribution of the eight 6-mer sequences (Fig. 4d) on N20U is described in Fig. 4f. Similar to the results in Fig. 4c, the first base positions for these 6-mer sequences were mainly situated in positions −17 to −9 in the TIR upstream of AUG, implying that in general the distance of the SD/anti-SD helix to the start codon varied from 3 to 11 bases (Fig. 4f). Interestingly, this relative position of the SD/anti-SD interaction on the 3′ end of the 16S rRNA was synergetic with the position of the SD sequence on mRNA. As shown in Fig. 4f, the peak of the regional preference on N20U was moving towards the start codon as the complementary positions at the 3′ end of 16S rRNA moved from 1 to 8. Combined with the crystal structure of the initiation complex^33^ (PDB code: 2QNH) (Fig. 4a), the synergetic effect implies that the steric assignment of the SD/anti-SD interaction in the mRNA entry tunnel can influence the energy landscape for conformational changes during the formation of the translation initiation complexes.

### Stability of mRNA secondary structure

**Figure 5:**
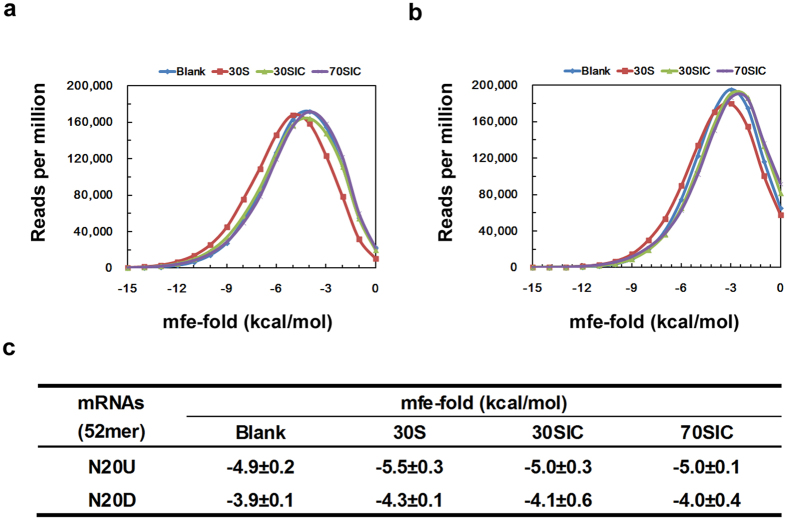
Statistical stability of mRNA secondary structures in the three initiation complexes. The stability was predicted as the minimum free energy for RNA folding using 52-mer full-length mRNAs. (**a**) Distribution of the minimum folding free energy for mRNA library N20U. (**b**) Distribution of the minimum folding free energy for mRNA library N20D. (**c**) Means of the minimum folding free energies for mRNAs recruited to initiation complex in three stages. The means were calculated from two independently repeated experiments.

Next, we analyzed the assembly of the mRNA secondary structure in different initiation complexes. For each library of N20U and N20D, the minimum free energy (mfe) for RNA folding was predicted for the 52-mer mRNAs in the blank control, 30S, 30SIC and 70SIC. Figure 5 compares the overall stability of the mRNAs recruited to different initiation complexes. For both N20U (Fig. 5a) and N20D (Fig. 5b), a slight shift towards a lower minimum free energy (mfe) of RNA folding was observed for 30S, which indicated a more stable secondary structure of the mRNAs chosen by the 30S subunit alone. For the mRNAs selected in 30SIC and 70SIC, the secondary structure of both N20U and N20D was statistically less stable. The average values of the minimum folding free energy were quantified (Fig. 5c), which further illustrated that the mRNAs recruited to the 30S subunit alone had more stable mRNA secondary structures.

## Discussion

For bacteria, three modes of translational initiation have been identified so far^34^. The best-known mode is the standard 30S-binding model, in which the small ribosomal subunit selects the initiation site on an mRNA with the help of three initiation factors. The other two modes are “leaderless mRNA” mode^35^,^36^ and “70S-scanning” mode^34^. This paper focused on the standard 30S-binding model. In the 30S-binding model, the ribosomes recruit mRNAs in a multiple-step processes by forming the 30S pre-initiation complex, the 30S initiation complex (30SIC), and the 70S initiation complex (70SIC). The small ribosomal subunit identifies the initiation codon (normally AUG) through the specific sequence signals located within its vicinity^13^,^14^,^15^. We attempted to dynamically study the initiation signals located at the translation initiation region by verifying the role of these signals during different initiation stages. Because the 30S pre-initiation complex is an intermediate state of the initiation, we used the 30S ribosome instead of the 30S pre-initiation complex, referred to as the 30S complex in our study.

Recognition of the mRNA by the ribosome is a dynamic process that occurs through modulation of the secondary structure at the translational initiation region (TIR) of mRNA^7^,^8^. In general, all mRNAs are structured to some extent, and the availability of initiation elements in the TIR of an mRNA for ribosome binding is affected by the stability of the mRNA secondary structure^9^. Structured mRNAs regulate the initiation of translation by competitively binding to the platform of the ribosome^37^. A widely accepted mechanism of this process is the “standby model”^8^,^17^. According to this model, the 30S ribosomal subunit binds rapidly and non-specifically to any single-stranded region of the mRNA. The mRNA structure and position on the 30S subunit would then restructure in a sort of induced fit to allow for a more specific interaction with the participation of initiation factors, fMet-tRNA^fMe^ and the 50S ribosomal subunit^1^,^11^,^16^. In this paper, we studied the mRNA identification during different stages of mRNA recruitment. Our results suggest a tendency for less structured mRNAs during translation initiation. Statistically, a lower assembled minimum free energy was observed for the mRNA folding in the 30SIC and the 70SIC compared with the 30S complex (Fig. 5). This finding is consistent with the prerequisite for the rapid binding during the formation of the pre-initiation complexes^8^. In the subsequent process, when the pre-initiation complex transfers to the initiation complex, structural rearrangement is required to bind mRNAs, and less structured mRNAs are then preferred. The less structured mRNAs can facilitate the exposure of the required initiation elements and ensure the proper placement of the mRNA around the neck of the 30S subunit^16^. Additional analysis supported the preference for less structured mRNA, such as the observation of the accumulated A-bases (Fig. 1) and the correlation of the second codon with the stability of the structured mRNAs (Fig. 2). Our observations agree well with the standby model.

Normally, the efficiency of mRNA recruitment is the cooperative and cumulative result of multiple initiation signals around the initiation codon^9^,^10^. Our results confirm the nature of these initiation signals at a global level. Using deep sequencing techniques, we identified the region recognized by S1 protein (Figs 1 and 3a), the Shine-Dalgarno (SD) sequence (Figs 1 and 3b), the second codon (Fig. 2), the effect of the distance between the SD sequence and the initiation codon (Fig. 4c,f), and the availability of the SD sequence for binding to the anti-SD (ASD) sequence near the 3′ end of the 16S rRNA (Fig. 4e). Furthermore, our data provide additional information on the regional specificity in the dynamic process of mRNA recruitment. The regional dynamics was inferred by the extent of the variation in local base preference during the different stages of initiation complex and may reflect the specific requirement for exposing different initiation elements through conformational changes during mRNA recruitment. The degree of the base preference changed significantly in the region where the SD/anti-SD interactions were dominant (Figs 1 and 3b), but not so much in the region corresponding to the interaction between the S1 protein and the AU-rich sequences (Figs 1 and 3a). Our results imply that the SD/anti-SD recognition is largely dependent on the mRNA conformational change during the formation of the initiation complexes. The synergetic effect observed in Fig. 4f sheds additional light on the importance of the conformational changes on the SD/anti-SD interaction during mRNA recognition. Moreover, the S1 interactions are less influenced by the conformational change, which might be because the 5′ end of an A/U-rich mRNA is less structured during translation initiation. However, the “anchoring point”^6^,^38^ of S1 is likely formed at the first stage of the 30S subunit recognition and is not affected in the subsequent structural reorganization as the initiation complex proceeds to the next stage. Moderate regulation was observed for other fractions of mRNA, such as the spacer sequences between the SD and start codon (Figs 1 and 3c), the +2 codon (Fig. 2c), and the sequence downstream of AUG (Supplementary Fig. S1), providing additional information influenced by mRNA conformational change.

In summary, we have systematically investigated the sequence preference of mRNA recruitment by deep sequencing techniques. Our results provide a comprehensive and dynamic view of the sequence preference in the TIR and have clarified and verified the conformational changes of the mRNA structure during translational initiation. In addition, more detailed information on the SD/anti-SD interactions and the second codon has been identified based on our statistical analysis. In living cells, mRNA recruitment is a kinetic phenomenon due to the competition between mRNAs and due to the kinetic checkpoints that occur after mRNA selection. Our current research could not circumvent the risk that the 30S initiation complex may not be stable; so, we must be cautious when utilizing the sequence information obtained under our experimental conditions. Moreover, our research was performed on translation initiation at the equilibrium state, presenting the thermodynamic, not kinetic, properties of the initiation complexes. However, the true translation efficiency is determined not only by how easily the initiation complex forms but also how quickly it can slip into the next elongation step. Thus, it would be helpful to further elucidate the fine-tuned relationship between the thermodynamic stability of the initiation complex and the dynamic feasibility of the subsequent mRNA translocation steps within the TIR of mRNA.

## Methods

### General methods and materials

All DNA oligonucleotides were from Sangon Biotech (Shanghai, China). Methionine (M9625), folinic acid (F7878), initiator tRNA from *E. coli* (R8019) and GTP (G8877) were purchased from Sigma (St. Louis, MO, USA). Ni-NTA agarose and the QIAquick PCR Purification Kit were obtained from Qiagen (Hilden, Germany). The Ampliscribe T7 High Yield Transcription Kit was ordered from Epicentre (Madison, WI, USA). The NEBNext Multiplex Small RNA Library Prep Set for Illumina was ordered from NEB (Ipswich, MA, USA). The HiTrap chelating HP column (5 mL) and Superdex 200 (120 mL) were purchased from Amersham Pharmacia Biotech Inc. (Piscataway, NJ, USA). Chromatography was performed using an Amersham Biosciences ÄKTApurifier equipped with Unicorn 4.10 software (Piscataway, NJ, USA). Sequencing was performed using an Illumina HiSeq 2000 system (San Diego, CA, USA).

The genes encoding *E. coli* methionyl-tRNA synthetase, methionyl-tRNA_i_^Met^ formyltransferase and the translation initiation factor proteins IF1, IF2 and IF3 were amplified from chromosomal DNA of *E. coli* BL21 (DE3) and subcloned into a pET28a vector. His-tagged proteins were overexpressed in *E. coli* and purified in by FPLC using a HiTrap chelating HP column followed by gel filtration using Superdex 200. Initiator fMet-tRNA_i_^Met^ was prepared as previously described^39^,^40^.

Ribosomes were purified from *E. coli* BL21 (DE3)^41^. Ribosomal proteins S6 and L11 were chromosomally engineered by adding N-terminal His-tags for purification of 30S ribosomes (Supplementary Fig. S9) and 70S ribosomes, respectively. The activity of the 70S ribosome was further verified by *in vitro* translation of YFP (Supplementary Fig. S10).

### Randomized mRNA library

The randomized mRNA library was prepared by *in vitro* transcription using an Ampliscribe T7 High Yield Transcription Kit with some modifications. Briefly, each DNA oligonucleotide (Supplementary Table S1) was hybridized with a T7 promoter (5′-TAATACGACTCACTATA-3′). *In vitro* transcription was carried out in a 50-μL (total volume) buffered reaction mixture containing 1 μM hybridized template DNA and 30 mM GMP. All the other components were the same as suggested by the kit. The reaction was incubated at 37 °C overnight. After digestion of the template DNA with RNase-free DNase, the mRNAs were purified by 7.0 M urea/15% PAGE. The bands were excised, and mRNAs were eluted overnight with elution buffer (0.5 M NaOAc, pH 5.0 and 0.1 mM EDTA). The final transcripts were then precipitated with ethanol, resuspended in 0.1 mM EDTA and stored at −80 °C.

### Recruitment of mRNAs

Randomized mRNAs (2.0 μM) were recruited from the ribosome at the different initiation complexes with 0.2 μM ribosomes (30S or 70S), either in the absence or presence of 0.4 μM IFs and 0.4 μM initiator tRNAs (Supplementary Fig. S1). Generally, the translation initiation was carried out in a 50- μL (total volume) buffered reaction mixture containing 50 mM Tris-HCl, pH 7.6, 70 mM NH_4_Cl, 10 mM MgCl_2_, 30 mM KCl, 1 mM DTT, 1.0 mM PMSF, 0.2 U/μL RNasin and 1.0 mM GTP, 2.0 μM mRNAs and 0.2 μM ribosomes, in the absence or presence of the 0.4 μM of IFs and initiator tRNAs. The reaction was incubated at 37 °C for 30 minutes for the initiation complex to reach equilibrium.

Initiation complexes were collected using Ni-NTA chromatography. The initiation reaction mixture (50 μL) was diluted with 450 μL of 50 mM Tris-HCl, pH 7.6, containing 70 mM NH_4_Cl, 10 mM MgCl_2_, 30 mM KCl, 1.0 mM DTT, 1.0 mM PMSF and 0.2 U/μL RNasin, and mixed gently with 250 μL of a 50% slurry of Ni-NTA resin for 16 hours at 4 °C. After centrifugation at 1600 rpm for 2 minutes, the resin pellet was washed once with 500 μL of 50 mM Tris-HCl, pH 7.6, containing 70 mM NH_4_Cl, 10 mM MgCl_2_, 30 mM KCl, 1.0 mM DTT, 1. mM PMSF and 0.2 U/μL RNasin. The initiation complex was eluted twice with 250 μL of 50 mM Tris-HCl, pH 7.6, containing 70 mM NH_4_Cl, 10 mM MgCl_2_, 30 mM KCl, 1.0 mM DTT, 1.0 mM PMSF, 0.2 U/μL RNasin and 150 mM imidazole. After elution, the collected complexes were combined and treated with phenol:chloroform to remove ribosomal proteins. Finally, the pools of RNAs were precipitated with ethanol, resuspended in distilled H_2_O, and used directly for sequencing library preparation. The RNA pools included both the ribosomal RNAs and the selected mRNAs binding to initiation complexes.

### Sequencing library preparation

The sequencing library was prepared by following the procedure described in NEBNext Multiplex Small RNA Library Prep Set for Illumina. Briefly, 6 μL of the RNAs (with an effective mRNA input approximately 10^12^ molecules per preparation) were ligated to 10x concentrations of the multiplex 3′ SR adaptor (5′-rAppAGATCGGAAGAGCACACGTCT-NH_2_-3′) and the 5′ SR adaptor (5′-rGrUrUrCrArGrArGrUrUrCrUrArCrArGrUrCrCrGrArCrGrArUrC-3′), followed by reverse transcription. The cDNA constructs were then amplified using PCR by modification of the extension time at 62 °C to 16 s and the number of cycles to 15. The PCR amplified cDNA constructs were purified using a QIAquick PCR Purification Kit followed by gel chromatography using 6% native PAGE (Supplementary Fig. S11). The bands of the proper size for the cDNA constructs were excised and eluted overnight with the elution buffer (provided by the kit). The final cDNA constructs were then precipitated with ethanol and resuspended in distilled H_2_O. Finally, 1.0 μL of 1.0 nM of the cDNA constructs (around of 6 × 10^8^ molecules) were used for deep sequencing (2G paired-end) on Illumina HiSeq sequencing platform.

### Data processing

Deep sequencing of randomized mRNA libraries recruited to ribosomes in the three initiation stages was sequenced using the Illumina HiSeq 2000 system. After sequencing, restrictive quality control was performed to remove the low-quality sequencing reads and possible contamination from degraded rRNAs. The final effective reads were described as the mRNA reads containing complete information in the randomized region (Supplementary Table S2).

### K-mer analysis for sequence properties

To describe the characteristics of the randomized region in N8U, N8M, and N8D selected by different initiation complexes, k-mer analysis was performed using Jellyfish (version 1.1.11)^42^,^43^. K-mer analysis^42^ means to output a 2D-histogram of the analyzed sequences scanned by a given length k (where k equals a positive integer) to a 2-D box containing all possible substrings of length k, i.e., when k = 1, the 2-D box would contain only 4 positions A, T.C, and G; when k = 2, the 2-D box will be 4^2^ = 16 containing all possible two-base sequences (AA, AT, TA, TT; AC, CA, CT, TC; AG, GA, GT, TG; CC, CG, GC, GG).

In our experiment, a k-mer analysis was performed to count the frequency of all the possible subsequences of length k (k = 6) for all reads of a sequencing library. The frequency of different k-mers reflected the characteristics of the randomized region enriched during our selection. For 6-mer sequencing, there are 4096 possible nucleotide combinations (equal to 4^6^). The k-mer count was evaluated as the sequence occurrence for each of the individual 6-mer sequences in the blank control (the mRNA library alone), 30S, 30SIC, and 70SIC. Because the library size was different in each sample, the k-mer counts for each individual sequence were normalized by dividing by the total k-mer counts of the sample itself, followed by multiplication by 1,000,000. We used k-mer counts per million reads (KPM) to represent this normalized k-mer occurrence. To deduct the influence of the background, the relative KPM (KPM_relative_) was further proceeded by comparison of the KPM of each 6-mer sequence in the selected samples (30S, 30SIC and 70SIC) with the KPM of the same 6-mer in the blank control of the same library. The following formula was used: KPM_relative_ = [lg(KPM_sample_ + 1) − lg(KPM_control_ + 1)]. The KPM_relative_ values of all the k-mer sequences were fitted into a 2-D geometric representation, where each k-mer sequence had a unique coordinate in a 2^k^ × 2^k^ matrix^42^. The sequence characteristics was then visualized as the KPM_relative_ pattern in a heat map using R version 3.2.3 (https://www.r-project.org/).

### Prediction of hybridization free energy

The hybridization free energy^44^ between the k-mer sequence (k = 6) and a 13-mer target sequence was predicted with RNAhybrid-2.1.2 (http://bibiserv.techfak.uni-bielefeld.de/rnahybrid/). The 13-mer target sequence was 5′-GAUCACCUCCUUA-3′, located at the 3′ end of the 16S rRNA.

### Stability of mRNA secondary structure

The stability of the mRNA secondary structure was predicted as the minimum free energy for RNA folding^45^. The RNAfold program in the Vienna RNA-2.1.7 package (http://www.tbi.univie.ac.at/RNA/) was used for this prediction. The input mRNAs were the full-length 52-mer mRNAs in each mRNA library of N20U and N20D.

## Additional Information

**Accession code:** The sequencing data discussed in this paper have been deposited in NCBI’s Gene Expression Omnibus and are accessible through GEO Series accession number GSE69782. These data can be accessed at the following link: http://www.ncbi.nlm.nih.gov/geo/query/acc.cgi?acc=GSE69782.

**How to cite this article**: Gao, R. *et al*. Deep sequencing reveals global patterns of mRNA recruitment during translation initiation. *Sci. Rep.* **6**, 30170; doi: 10.1038/srep30170 (2016).

## Supplementary Material

Supplementary Information
