# Supplementary material for: Deep sequencing reveals global patterns of mRNA recruitment during translation initiation
Source: Sci Rep. 2016 Jul 27;6:30170. doi: 10.1038/srep30170 (PMC4962057; doi:10.1038/srep30170)
Supplement: Supplementary Information [file srep30170-s1.pdf]

Supplementary information for:

Deep sequencing reveals global patterns of mRNA recruitment during translation initiation

Rong Gao[1#*], Kai Yu[1#], Ju-Kui Nie[1],Teng-Fei Lian[1], Jian-Shi Jin[1], Anders Liljas[2] and Xiao-Dong Su[1*]

[1] Biodynamic Optical Imaging Center (BIOPIC), and State Key Laboratory of Protein and Plant Gene Research, School of Life Sciences, Peking University, Beijing 100871, China
[2] Department of Biochemistry and Structural Biology, Lund University, Lund, Sweden

SUPPLEMENTARY DATA

Table S1. DNA oligonucleotides synthesized for *in vitro* transcription of mRNAs libraries

Oligos	Sequence
**N20U**	5′-TTTTTTTGTTTCCCGTTGAATATGGCTCAT*NNNNNNNNNNNNNNNNNNNN*CCTATAGTGAGTCGTATTA-3′
**N20D**	5′-TTTTTTT*NNNNNNNNNNNNNNNNNNNN*CATAATATACCTCCTTAATTTTTCCTATAGTGAGTCGTATTA-3′
**N8U**	5′-TTTTTTTGTTTCCCGTTGAATATGGCTCATAATATACCTCCT*NNNNNNNN*CCTATAGTGAGTCGTATTA-3′
**N8M**	5′-TTTTTTTGTTTCCCGTTGAATATGGCTCATAATATA*NNNNNNNN*ATTTTTCCTATAGTGAGTCGTATTA-3′
**N8D**	5′-TTTTTTTGTTTCCCGTTGAATATGGCTCAT*NNNNNNNN*TCCTTAATTTTTCCTATAGTGAGTCGTATTA-3′

Table S2. The effective total reads used for analysis of the mRNAs recruited to initiation complex at different stages after deep sequencing

	**N20U**	**N20D**	**N20U'**	**N20D'**	**N8U**	**N8M**	**N8D**
**Blank**	3,537,137	5,964,381	389,038	1,359,386	525,379	1,934,143	774,305
**30S**	380,366	2,568,078	25,360	267,916	127,788	207,235	789,359
**30SIC**	1,521,384	2,606,090	99,396	196,692	68,900	251,373	159,304
**70SIC**	1,014,606	4,788,969	289,217	335,163	360,883	263,846	965,785

Note: N20U’ and N20D’ means independently repeat of the experiments for N20U and N20D.

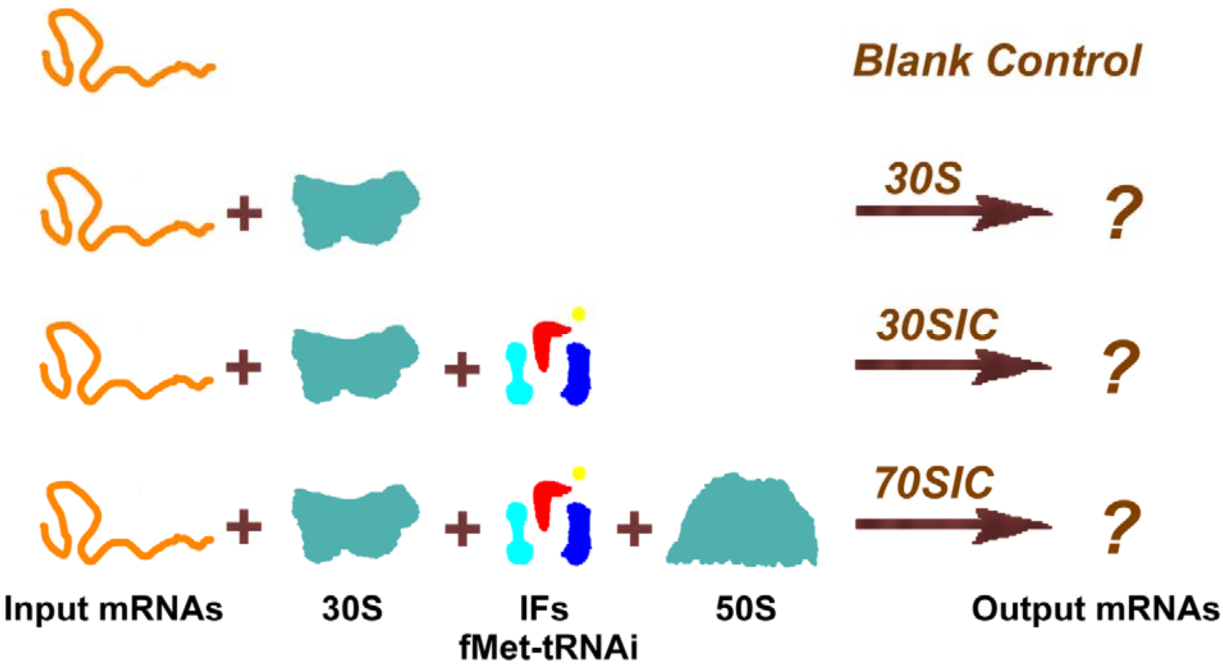

Figure S1. Schematic of experimental design in study of recruitment of mRNAs in formation of translational initiation complex at different stages.

mRNA-reference: GGAAAAAUUAAGGAGGUAUAUU<u>AUG</u>AGCCAUAUUCAACGGGAAACAAAAAAA

-20 -15 -10 -5 -1 0 +1 +5 +10 +15 +20

mRNA-N20U: GG*NNNNNNNNNNNNNNNNNNNN*<u>AUG</u>AGCCAUAUUCAACGGGAAACAAAAAAA

-20 -15 -10 -5 -1 0 +1 +5 +10 +15 +20

mRNA-N20D: GGAAAAAUUAAGGAGGUAUAUU<u>AUG</u>*NNNNNNNNNNNNNNNNNNNN*AAAAAAA

-20 -15 -10 -5 -1 0 +1 +5 +10 +15 +20

mRNA-N8U: GG*NNNNNNNN*AGGAGGUAUAUU<u>AUG</u>AGCCAUAUUCAACGGGAAACAAAAAAA

-20 -15 -10 -5 -1 0 +1 +5 +10 +15 +20

mRNA-N8M: GGAAAAAU*NNNNNNNN*UAUAUU<u>AUG</u>AGCCAUAUUCAACGGGAAACAAAAAAA

-20 -15 -10 -5 -1 0 +1 +5 +10 +15 +20

mRNA-N8D: GGAAAAAUUAAGGA*NNNNNNNN*<u>AUG</u>AGCCAUAUUCAACGGGAAACAAAAAAA

-20 -15 -10 -5 -1 0 +1 +5 +10 +15 +20

Figure S2. Messenger RNA libraries containing randomized nucleotides in TIR.

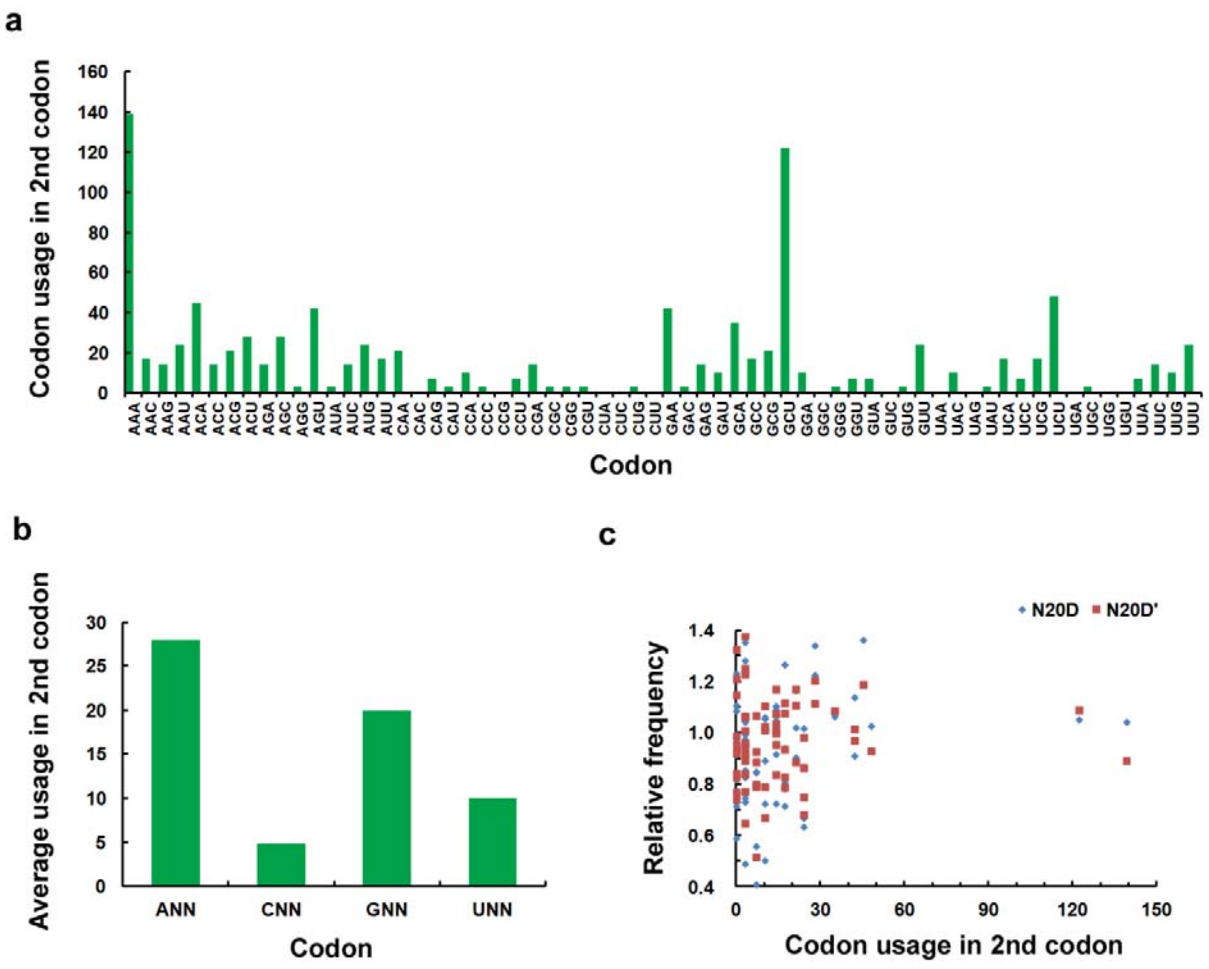

Figure S3. Codon preference for the second codon directly following AUG initiation codon. (a) Codon usage in the second position from the literature[19]. The codon usage presented here is the average usage as the second codon per 1000 genes calculated from 288 genes[19]. (b) The average usage in the second position for codons ANN, CNN, GNN and UNN. The average usage was the average of the codon usage in the second codon (ref. the definition in Supplymentary Figure S4a) for codons starting with A, C, G or U, respectively. (c) Correlation between the codon preference in the 70SIC complex and the codon usage from the literature [19] in the second codon for all the 64 codons.

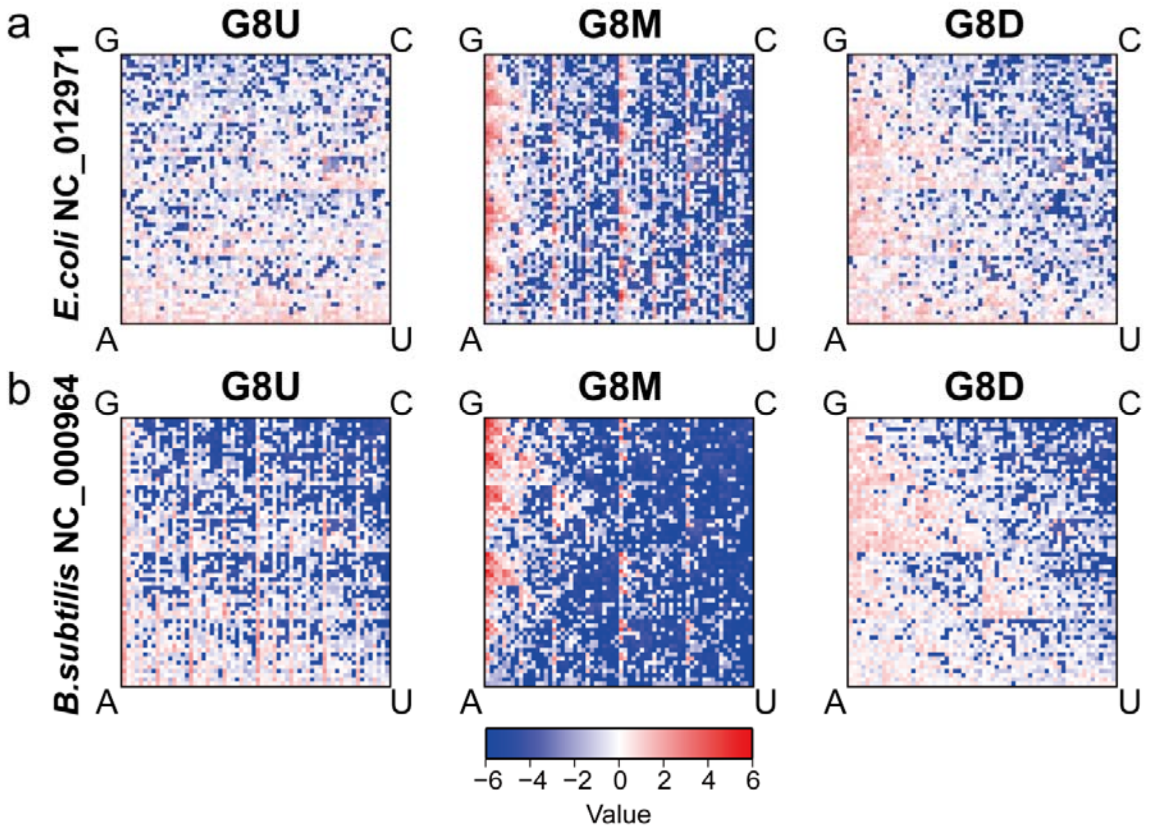

Figure S4. Genome analysis of the regional characteristics of the sequence at TIR upstream of the ORF. The k-mer analysis (k = 6) was performed for (a) the gram-negative strain of *E. coli* NC_012971 and (b) the gram-positive strain of *B. subtilis* NC_000964. G8U, G8M, and G8D are corresponding to the same regions as those described in randomized mRNA library N8U, N8M, and N8D, respectively.

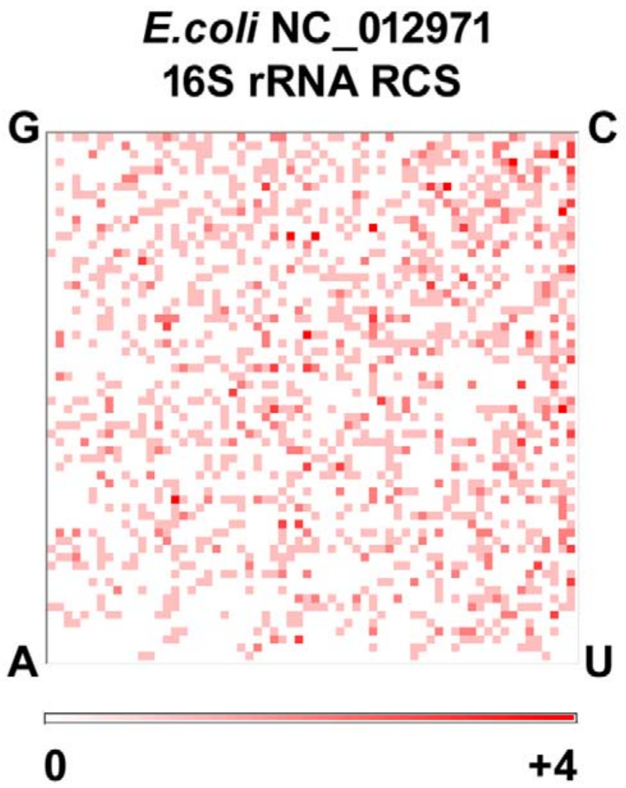

Figure S5. Gene Pattern represented by kmer analysis (k = 6) of the reverse complementary sequence of the 16S rRNA from *E.coli* NC_012971 (16S rRNA RCS). The color bar shown below demonstrated the abundance of the 6-mer sequences presented in the 16S rRNA RCS.

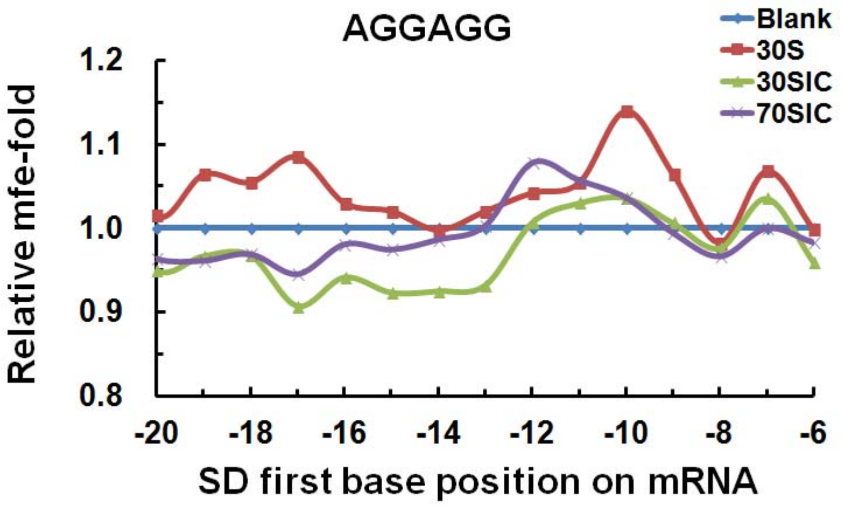

Figure S6. Correlation of the relative positions of AGGAGG on N20U and the statistic stability of their corresponding mRNAs. The stability of mRNAs was predicted statistically as the means of the minimum RNA folding free energy for 52-mer full length mRNAs containing AGGAGG at the same position. The relative stability was calculated as the normalized mfe-fold devided by those from blank control. When the value of relative mfe-fold higher than one, the secondary structure of the mRNAs was statistically considered more stable when compared with those observed in the blank control.

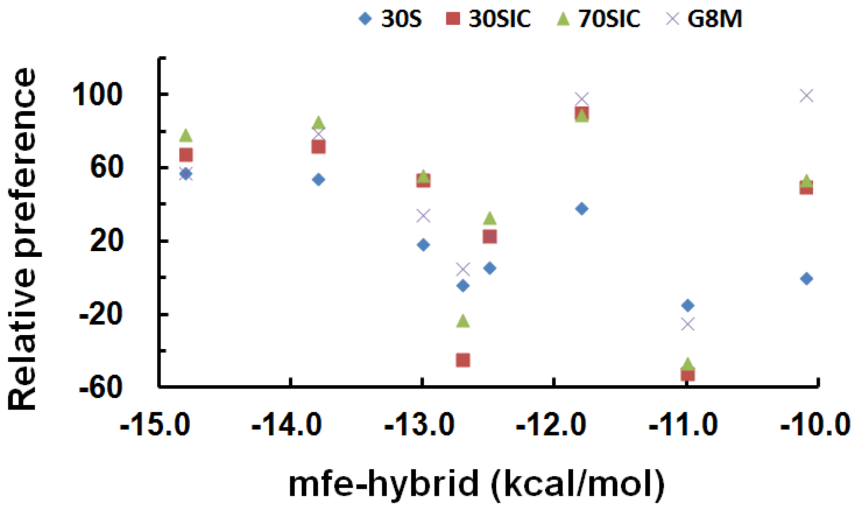

Figure S7. Correlation of the sequence preference with their binding affinity to 3'end of 16S rRNA. The sequences were the eight 6-mer sequences shown in Figure 4d (orange color). The binding affinity was predicted as the minimum free energy for these sequences hybridized to 3' end of 16S rRNA in *E. coli* shown in Figure 4d (black color).

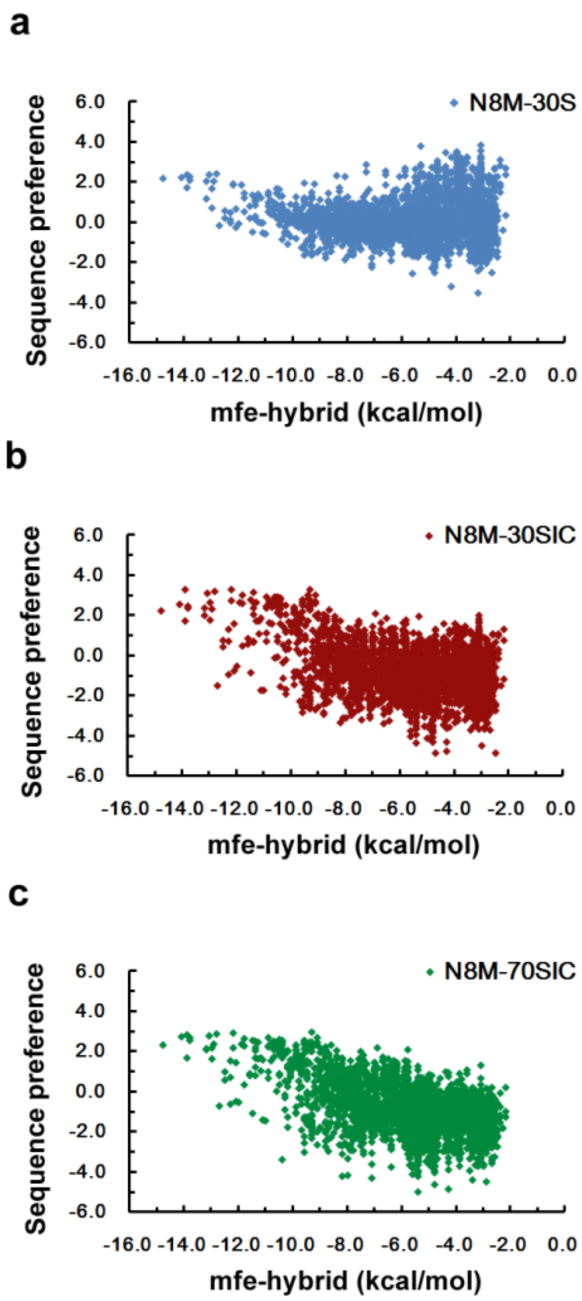

Figure S8. Statistic correlation of the sequence preference with their binding affinity to the 3' end of 16S rRNA. The binding affinity was predicted as the minimum free energy for all the possible 6-mer RNA sequences, which might hybridize to any position on the 13-mer at the 3' end of 16S rRNA. The sequence preference was described as in (a) 30S, (b) 30SIC and (c) 70SIC.

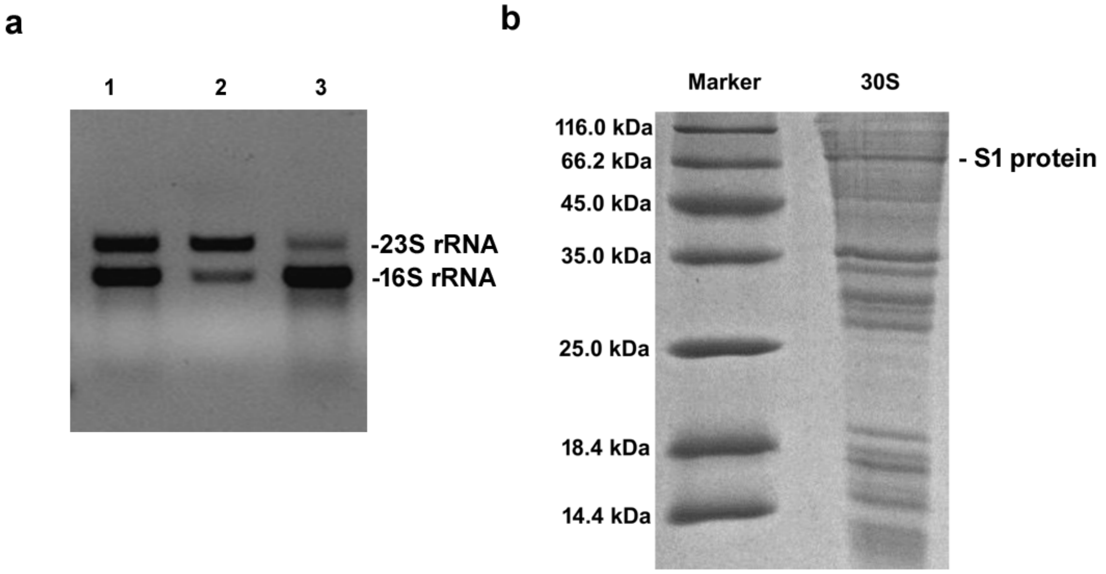

Figure S9. Isolation of 30S ribosome from 70S ribosome using FPLC. The 70S ribosome labeled the N-terminal of S6 with His-tag was purified as described in the publication[41] using high Mg^{2+} buffer containing 20 mM Tris-Cl, pH7.6, 10 mM $MgCl_2$, 150 mM KCl, 30 mM NH_4Cl and 150 mM imidazole. Then the 30S ribosome was isolated from the purified 70S ribosome using low Mg^{2+} buffer containing 20 mM Tris-Cl, pH7.6, 1 mM $MgCl_2$, 150 mM KCl, and 30 mM NH_4Cl. (a) Verification of the purification process using 1% agarose gel. Lane1, 70S ribosome loaded on the Ni^{2+} column; lane2, flow-through from Ni^{2+} column containing mainly 50S ribosome; lane3, elution from Ni^{2+} colume with 150 mM imidazole containing mainly 30S ribosome. (b) Verification of S1 protein in the isolated 30S ribosome using 18% SDS-PAGE. The indicated band had been further confirmed as S1 protein using N-terminal protein sequencing.

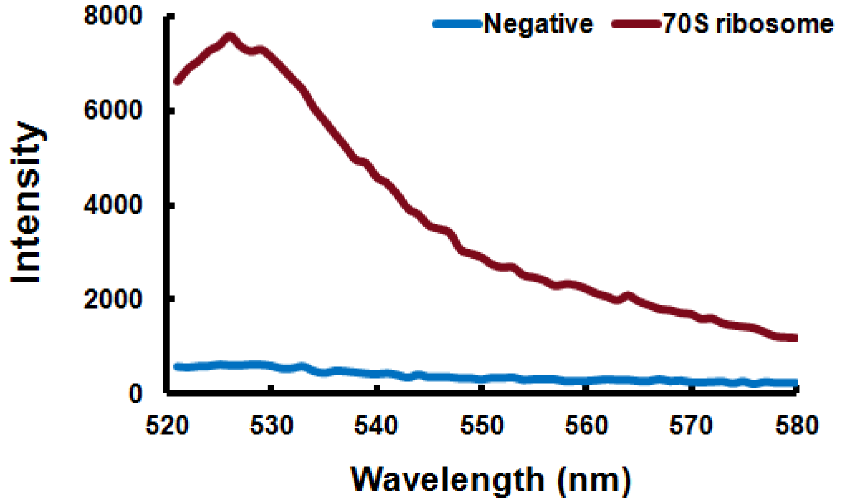

Figure S10. Verification of the activity of the purified 70S ribosome. The *in vitro* translation reaction was carried out in 6μL reaction mixture containing 120 ng plasmid encoding for YFP either without ribosome as negative control or with 0.2 μM of purified 70S ribosome. The reaction was incubated @37°C for 1 h and then diluted 250 times. The yield of YFP was measured by fluorescence with excitation at 510 nm and emission from 520nm to 580 nm.

a

b

c

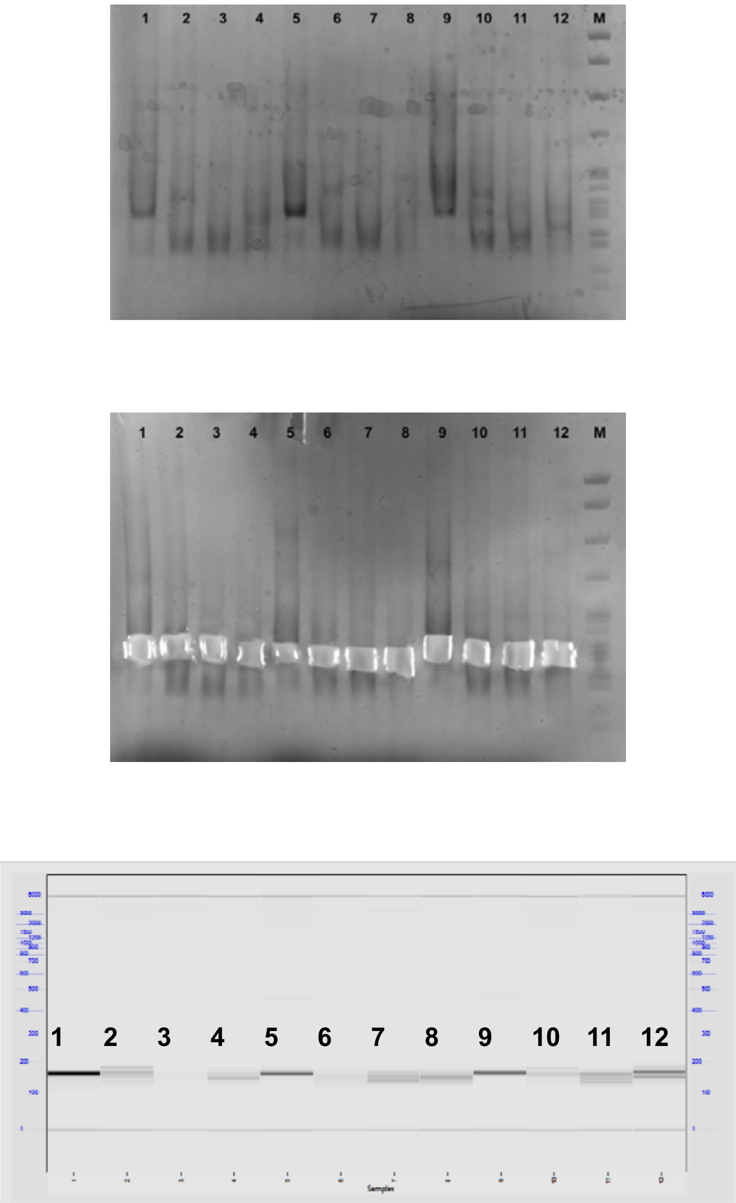

Figure S11. Representative information on the gel purification of the sequencing cDNA constructs. The PCR amplified cDNA constructs were purified using a QIAquick PCR Purification Kit followed by gel chromatography using 6% native PAGE. (a) and (b) Selection of the proper sequencing cDNA constructs by cutting the bands from the gel. (c) Quality control of the isolated cDNA constructs using Fragment Analyzer. Lanes 1-4, the libraries for N8U; lanes 5-8, the libraries for N8M; and lanes 9-12, the libraries for N8D. Lanes 1, 5 & 9, the control libraries; lanes 2, 6 & 10, the libraries isolated from 30S; lanes 3, 7 & 11, the libraries isolated from 30SIC; lanes 4, 8 & 12, the libraries isolated from 70SIC.
